# An Efficient Multilayer Approach to Model DNA-Based Nanobiosensors

**DOI:** 10.1021/acs.jpcb.2c07225

**Published:** 2023-02-13

**Authors:** Jesús Lucia-Tamudo, Juan J. Nogueira, Sergio Díaz-Tendero

**Affiliations:** † Department of Chemistry, Universidad Autónoma de Madrid, 28049, Madrid, Spain; ‡ Institute for Advanced Research in Chemistry (IAdChem), Universidad Autónoma de Madrid, 28049 Madrid, Spain; ¶ Condensed Matter Physics Center (IFIMAC), Universidad Autónoma de Madrid, 28049 Madrid, Spain

## Abstract

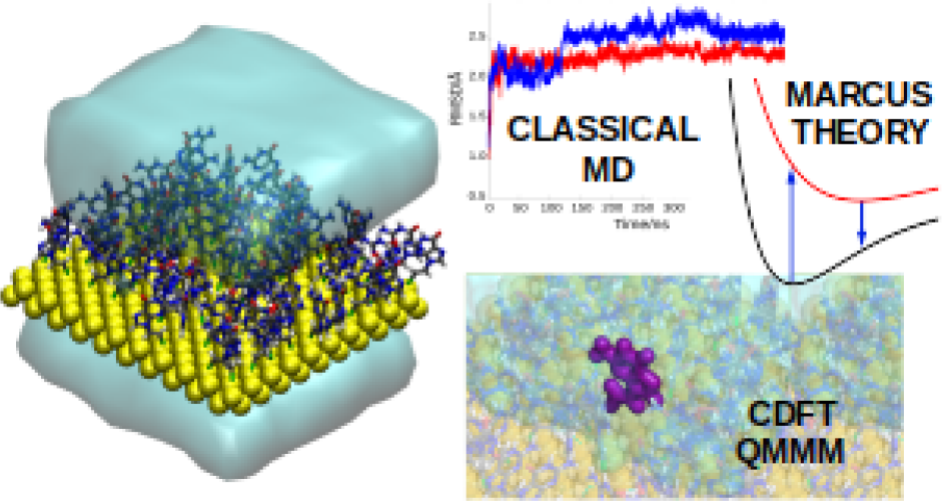

In this work, we present a full computational protocol to successfully obtain the one-electron reduction potential of nanobiosensors based on a self-assembled monolayer of DNA nucleobases linked to a gold substrate. The model is able to account for conformational sampling and environmental effects at a quantum mechanical (QM) level efficiently, by combining molecular mechanics (MM) molecular dynamics and multilayer QM/MM/continuum calculations within the framework of Marcus theory. The theoretical model shows that a guanine-based biosensor is more prone to be oxidized than the isolated nucleobase in water due to the electrostatic interactions between the assembled guanine molecules. In addition, the redox properties of the biosensor can be tuned by modifying the nature of the linker that anchor the nucleobases to the metal support.

## 1 Introduction

**Figure 1:**
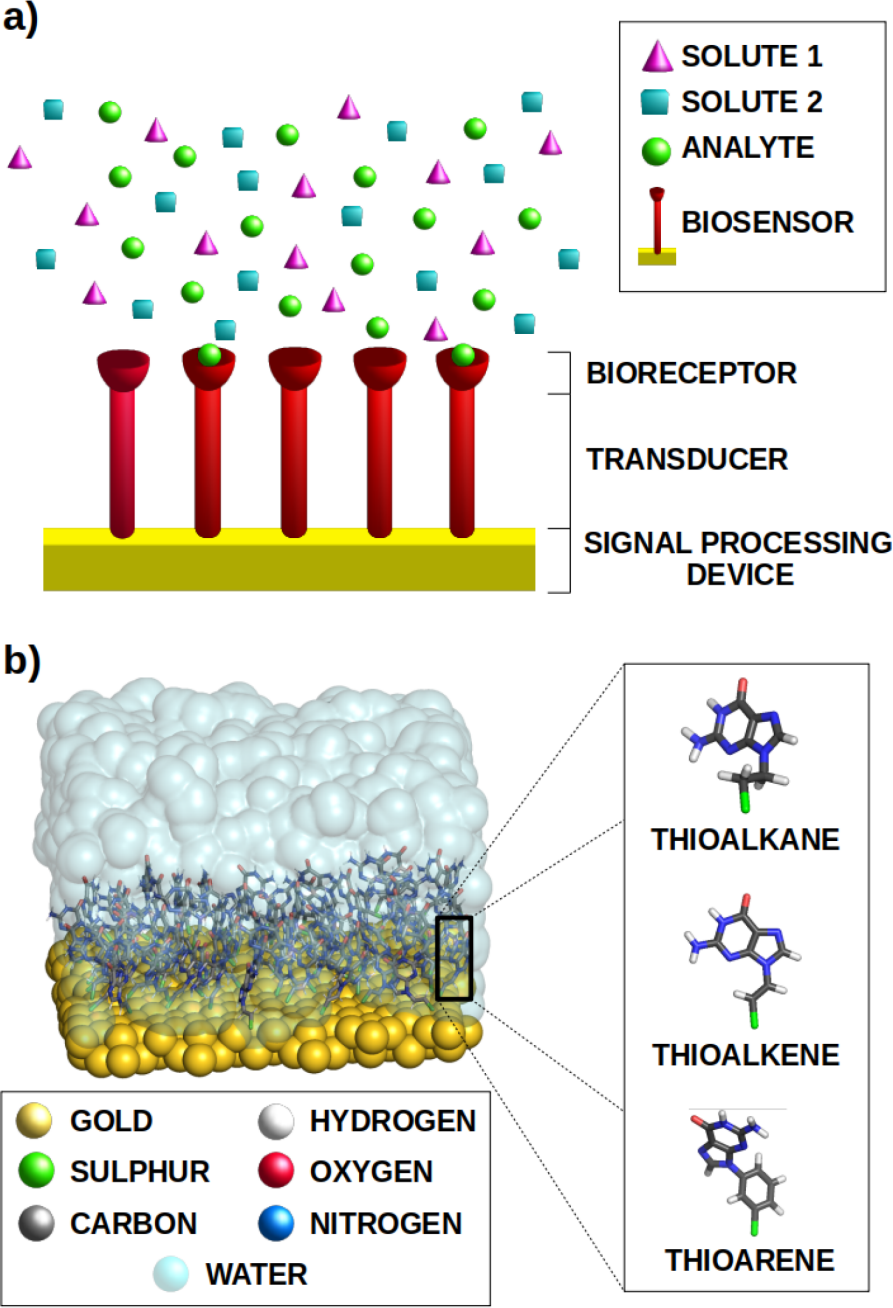
Schematic representation of (a) the general structure of a standard biosensor, and (b) the models employed in this study: An ensemble of guanines anchored to a gold surface by different organic linkers. The coverage of the gold surface sites by the guanine ligands was chosen to be 70%.

Biosensors are currently one of the most powerful tools for detecting the presence of specific analytes and determining their concentrations in a given sample.^1,2^ In fact, biosensors are widely used in many fields, such as health service,^2−10^ control assurance,^1,11−14^ environmental science,^2,15^ biology,^16,17^ and many others. These analytical devices are able to convert a biochemical signal, e.g., modification of the levels of a biospecies of interest, into a measurable signal. Generally, their operation mode is the following (see Figure 1a). First, the analyte is trapped by the bioreceptor due to chemical or physical interactions. Then, the raw signal is sent to the transducer and transformed to an appropriate signal to be read by the signal processing device. The nature of this measurable signal can be electrochemical,^18−20^ magnetic,^21−23^ optical,^24−27^ piezoelectric,^16^ or thermal,^2^ among others. Furthermore, the intensity of the signal must be proportional to the amount of analyte in the sample to obtain adequate detection.

Because of the wide range of constituents, in terms of their nature and the selected analytical technique for the detection, there is a huge amount of possible combinations to design a biosensor.^2^ Focusing on the bioreceptor, the key point for its selection resides in the fact that it cannot lose its properties once it is integrated into the sensor. Otherwise, the analyte could not interact with the bioreceptor as it usually does, when the latter is in its free form. In addition, the bioreceptor must be chosen selectively in order to respond only to one specific analyte or one class of analytes. Bioreceptors typically employed are enzymes, immunosensors, bioreceptors based on polymers, DNA or nucleobases, tissues, or even organelles.^2−4,15−17^

Electrochemical techniques are the most popular choice to measure the transformed signal for the detection task, because of their low cost, portability, simplicity of construction, straightforward use, high sensibility, and selectivity.^19,20,28−33^ Within this group of devices, there is a large variety of methods that can be employed for signal processing: voltammetry, amperometry, potentiometry, conductimetry, spectroscopy of electrochemical impedance, and so on. Recently, electrochemical DNA-based biosensors have become a convenient choice when trying to detect specific sequences of nucleic acids, heavy metal ions, and organic molecules, as well as using them as nanowires.^34−41^ This type of biosensor consists of an ensemble of single-stranded DNA (ss-DNA) or double-stranded DNA (ds-DNA) molecules adsorbed on a metal surface, constituting a self-assembled monolayer (SAM).^42−45^ Several issues must be addressed when designing and employing these devices. First, the nucleobase is the main moiety responsible for the electron transfer along the DNA strand, and thus, intense research has been aimed to obtain accurate redox properties of nucleobases to understand and characterize the charge-transfer processes that occur on the transmission of the signal along the DNA strand.^46−58^ In this context, guanine is the most susceptible nucleobase to be oxidized. Second, the most common metals employed as substrates of SAMs are Au, Ag, Cu, Pt, Hg, Ga, and As. However, not only the nature of the metal but also its crystallographic structural organization plays an important role.^59^ In the case of gold surfaces, the most popular and stable crystal orientations are the Au(100) and Au(111) ones. Finally, the interaction between the biosensor and the metal is an important issue. For example, most of the studied SAMs are constituted by a gold substrate covalently bonded to thiolated or selenated organic molecules, since the Au–S(Se) bond is very strong and stabilizes the assembly.^60−62^

Along this study, the one-electron reduction potential of a SAM biosensor formed by guanine molecules anchored to a Au(100) surface by three different linkers, namely, thioalkane, thioaryl, and thioalkene species (see Figure 1b), is investigated by means of computational methods. We will use the term “one-electron oxidation potential” as the reduction potential of an oxidation process (see (eq 1)). Specifically, the variation of the reduction potential of guanine is analyzed when going from the free nucleobase in solution to the SAM assembly. It was found that guanine is more prone to be oxidized when it is part of the SAM and, thus, the redox properties are enhanced for biosensing purposes. Moreover, it was determined that the selection of the linker can modulate the one-electron reduction potential of the biosensor. From a computational point of view, a protocol to obtain accurate one-electron reduction potentials for SAMs at affordable computational cost was developed and applied.

## 2 Methods

In the present work, two different types of protocols have been applied to compute the one-electron reduction potentials, depending on the size and complexity of the systems investigated. A static approach was applied when describing the reduction potentials of a guanine-linker complex in water, while a dynamic approach was employed when dealing with solvated self-assembled monolayers (SAMs). Both methodologies are shortly explained below.

### 2.1 Static Calculations by the Direct Approach

Given a reduction half-reaction as the one shown in eq 1,
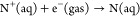
1the reduction free energy of this process can be written as
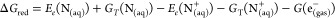
2where the free energy of each single species is an additive contribution of the electronic energy *E*_*e*_ and the thermal correction to the Gibbs free energy *G*_*T*_, which accounts for translational, rotational, vibrational and electronic terms. *G*(*e*_(gas)_^–^) accounts for the free energy of the electron. The terms *E*_*e*_ and *G*_*T*_ for each species participating in eq 1 are computed at their corresponding optimized geometries in a continuum solvent model as it is the Conductor-like Screening model (COSMO).^63−65^ It is remarkable to comment that this procedure will be only applied to the free organic molecules that will be later inmobilized on the SAM.

The reduction potential of a redox half-equation *E*_red_ is associated with the free energy of the process as follows:
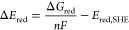
3where *F* is the Faraday constant, *n* is the number of exchanged electrons and *E*_red,SHE_ is the reduction potential of a reference electrode, which, in this case, is the standard hydrogen electrode (SHE). The considered value of *E*_red,SHE_ was 4.281 V, used in previous works.^66−70^ This value also accounts for the free energy of the electron in the gas phase using the Fermi–Dirac statistics, *G*(*e*_(gas)_^–^) = −0.867 kcal/mol.^71−73^ As a result, this contribution must also added in eq 2.

In order to assess the one-electron oxidation potential of organic molecules in which the positive charge of the cationic species is localized within the guanine fragment, some restrictions must be imposed to fulfill this requirement. Dederichs et al.^74^ first formulated the basis of a technique called Constrained Density Functional Theory (CDFT). In this formalism, the Lagrange multipliers are used to constrain some observables as the charge or the spin within a user-selected region of the target molecule.^75^ In this context, it is possible to study charge-restrained systems as the ones studied in this work using CDFT.^76,77^

### 2.2 Dynamic Calculations by the Marcus Theory

When the size of a system is large, there is not a clear minimum along the potential energy surface (PES) but many accessible local minima can be populated. As a result, it is necessary to explore all these conformations to obtain an accurate value of of the properties of the system. This implies performing a sampling procedure in the theoretical model. In this study, there is a considerably increase of the size of the system when an ensemble of ligands forms a SAM using a gold surface as substrate with respect to the case in which a single ligand is free in aqueous phase. Thus, when the SAM is studied a dynamic protocol is needed to accurately describe the properties of such a system.

When conformational motion is introduced in the model, the thermal correction shown in eq 2 is not explicitly computed. Despite this, it can be indirectly approached considering an ensemble of conformations along the PES and averaging the desired property over all of them. An alternative formulation used in many theoretical works is the Marcus theory,^78−83^ which has been applied to compute the redox properties of a wide range of systems.^58,84,85^

Assuming that the solvent response is linear with respect to a change in the solute, it can be shown that the free energy can be expressed as follows:^86,87^
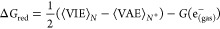
4where VIE stands for the vertical ionization energy and VAE represents the vertical attachment energy. The subscripts of the brackets indicate the phase space where the average value of the corresponding energetic term is computed. Thus, the VIE is computed in the phase space of the neutral system *N*, while the VAE is computed taking the PES of cationic system *N*^+^ as the reference. By running classical molecular dynamics (MD) simulations, the ensemble averages for each of the species can be easily calculated. First, several snapshots are randomly selected and the VIE (VAE) is computed for each frame by quantum mechanics/molecular mechanics (QM/MM) or QM/continuum approaches.^88^ The target region is included in the QM region, while the environment is described in terms of a MM force field or a continuum solvent model. Finally, the VIEs (VAEs) values are averaged.

The application of the Marcus theory is bound to some restrictions: (i) the distributions of the VIE and VAE must show a Gaussian shape, (ii) the standard deviations σ_VIE_, σ_VAE_ must be the same, and (iii) the reorganization energy (λ) must be equal to the variance of the VIE and VAE divided by a thermal factor of 2·k_B_·T, respectively.^85^ If these circumstances are not fulfilled, the Marcus theory suffers deviations. Nevertheless, these issues can be overcome using the quadratic model developed by Matyushov and Voth.^89,90^ This correction was applied to the systems that did not accomplish the requirements for employing the Marcus theory in this work.

## 3 Computational Details

### 3.1 Static Calculations for Solvated Guanine–Linker Complexes

All the QM/continuum and QM/MM calculations for the neutral and the cationic forms in aqueous phase were performed using the NWChem package.^91^ These calculations were performed using the PBEOP functional,^92−94^ which provided previously accurate results for nucleobases.^58,95^ The selected basis set was 6-311G(d)^96,97^ for all the atoms but for Au atoms, modeled with the LANL2DZ^98−100^ basis set and with the corresponding effective core potential (ECP), in order to include relativistic effects on those atoms. In the case of the cationic calculations, CDFT^101^ was applied in order to restrain the positive charge in the guanine moiety. The aqueous solvent in the QM/continuum calculations was described by COSMO.^63,64^ A frequency calculation was performed for each geometry to ensure that an energy minimum was reached and to compute the zero-point energy, which is part of the thermal correction.

### 3.2 Dynamic Calculations for Solvated SAMs

Classical MD simulations^102−104^ were run with the AMBER20 package^105,106^ and the systems were built up with AmberTools21^107^ and different homemade scripts that would be explained below. For both the cation and the neutral forms of each organic molecule, a set of force field parameters was developed, based on quantum mechanic calculations performed with the PBEOP functional, by the following procedure. First of all, the Hessian matrix for the optimized geometries obtained in the static calculations in aqueous phase was computed. Bond and bond angle parameters for the ligands were obtained from the Hessian matrix by the Seminario method, using PBEOP/6-311G(d).^108^ Parameters for dihedral angles, improper torsions, and Lennard-Jones nonbonded terms were taken from the generalized Amber force field (GAFF),^109^ with the exception of the nonbonded parameters of the Au atoms, that were taken from the literature.^110^ Electrostatic potential (ESP) charges were obtained from a PBEOP/6-31G(d) calculation in the aqueous phase. Each SAM was solvated in a tetragonal simulation box of ∼41 Å × 41 Å × 45 Å with 1441 water molecules, which were described with the TIP3P solvation model.^111^ For the SAM that holds a cationic organic molecule, a Cl^–^ anion was also added to neutralize the system, described by the Joung and Cheatham parameters.^112^

After the setup of the different systems, the same dynamic protocol was followed for all of them. The motion of the S and Au atoms is restrained by a force constant of 50 kcal/mol for the entire protocol, because a Au–S bonded interaction is not present in the force field. First, the system was minimized for 10 000 steps in which the steepest descent algorithm^113^ was used for the first 5000 steps and the Newton–Raphson algorithm^114^ was used for the last 5000 steps. After that, a constant volume (NVT) progressive heating to 300 K was performed for 500 ps. The Langevin thermostat was applied to control the temperature with a collision frequency of 2 ps^–1^. Then, an additional 500 ps simulation was run at 300 K in the NVT ensemble. Afterward, a 1 ns simulation was run in the NPT ensemble to balance the volume of the system and reach the correct density. Finally, a 500 ns production simulation was run in the NPT ensemble with the CUDA version of pmemd.^115,116^ The Berendsen barostat with anisotropic position scaling and a pressure relaxation time of 2 ps was employed to maintain the pressure constant at 1 bar. An interface in the *xy*-plane was established by applying an anisotropic pressure on this plane. During the full protocol, the particle-mesh Ewald method^117^ with a grid spacing of 1.0 Å was used to compute the electrostatic interactions and a 10 Å cutoff for the nonbonded interactions was chosen. The SHAKE algorithm^118−120^ restrained the bonds involving H atoms and a time step of 2 fs was used during the heating, equilibration, and production stages.

For each cationic and neutral trajectory of the SAMs, a specific number of snapshots were fetched randomly from the last 350 ns of the production trajectories. In the case of the snapshots of the neutral species, the VIEs were computed by running QM/MM and QM/MM/COSMO calculations, which represent examples of electrostatic and polarizable embedding, respectively. For the QM/MM/COSMO calculations, the explicit solvent molecules were removed from the different snapshots and replaced by COSMO, while the part of SAM that was not in the QM region was described by a force field. Regarding the cationic trajectories, the VAEs were computed also by QM/MM and QM/MM/COSMO calculations in the same way and introducing CDFT in the case of the cationic version of the SAM. In all the calculations, the QM region was described by the PBEOP functional and 6-311G(d) and LANL2DZ basis sets using NWChem. All these calculations were combined as explained in the previous section to obtain the one-electron oxidation potentials for the different studies performed.

### 3.3 Setup of the SAMs

**Figure 2:**
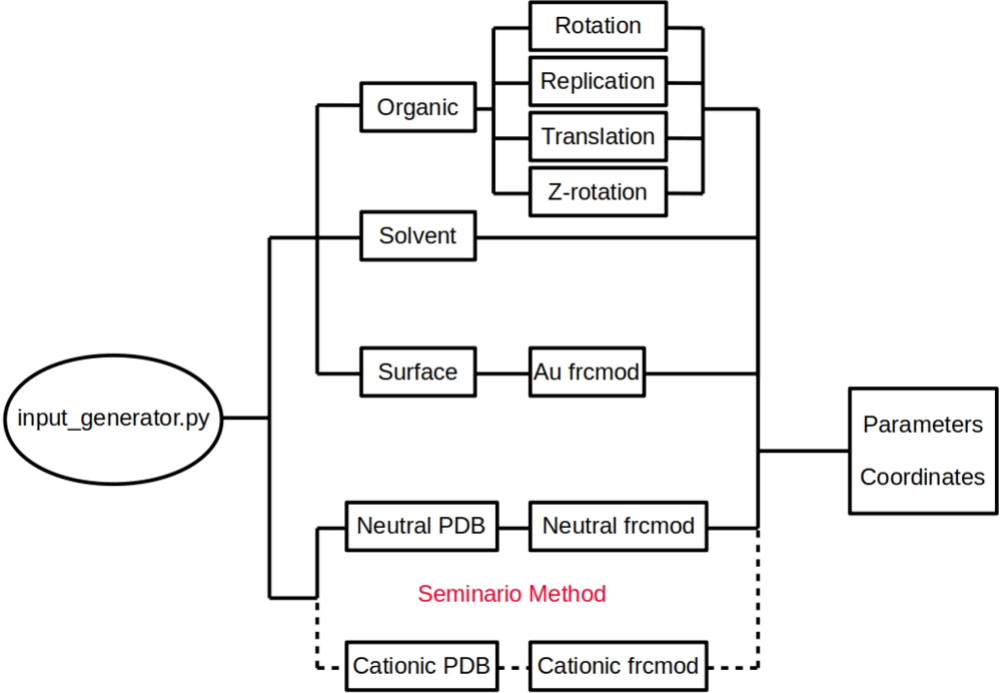
Schematic representation of the working principle of the algorithm to build up the SAM.

To perform the work presented in this manuscript, the system had to be built up using the following procedure. First of all, a model system was obtained from the CHARMM-GUI database.^121^ This tool provided a SAM in which an ensemble of tertiary thioalkanes was adsorbed onto a two-layer gold surface composed by 400 atoms. The coverage of the SAM was chosen to be 70%, and the entire system was solvated in water. This model was used as an starting guess. A python code was developed in order to modify this model to obtain the desired SAM in which each terciary thioalkane was replaced by a target organic molecule, namely, guanine and a linker (see Figure 2).

The PDB file of the target organic molecule is obtained from the NWChem output of the geometry optimization. In the case of the cationic SAM, both the neutral species and the cation are required and both PDB files are generated. Second, the code creates and run a calculation for each species to obtain the Hessian and the ESP charges associated with the molecule using the NWChem package. If the user provides these files, this step is skipped. Afterward, the Seminario method is employed to obtain the bond and bond angles parameters of the force field for the (cationic) organic ligand, while the rest of parameters are taken from the GAFF^109^ force field. Thus, all the parameters required for the setup of the system are obtained.

Then, the code splits the system into the three fundamental components: solvent, metal surface, and organic SAM. This step is performed to prepare each fragment to be properly read by tleap in order to generate the parameter and coordinate files for the Amber package. For the gold surface, the gold parameters are included. The interactions between the surface and the S atom of the ligands will be treated as nonbonded interactions and the position of both the Au atoms and the S atoms will be constrained along the dynamics by a force constant of 50 kcal/(mol Å^2^).

In the case of the organic part, the first step is to align the desired ligands with the ligands of the model system provided by CHARMM-GUI. An average tilt vector of the ensemble of tertiary thioalkanes of the model is obtained as well as a tilt vector of the target molecule. This second tilt vector is rotated so that it overlaps the tilt vector of the model to obtain a similar orientation of the target molecule with respect to the model molecules. This can be achieved using a rotation matrix **R**, provided by the Rodrigues formula,^122^ which requires the axis **u** = (*u*_*x*_, *u*_*y*_, *u*_*z*_) and the angle θ of rotation:
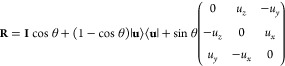
5The axis **u** = (*u*_*x*_, *u*_*y*_, *u*_*z*_) can be obtained performing a vectorial product between the two tilt vectors, and the angle is computed through the scalar product of these tilt vectors. Thus, the target molecule (or molecules in the case of the cationic SAM) can be rotated by applying eq 6 to all the atoms:
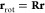
6Then, the rotated molecule is replicated and placed where each tertiary thioalkane was located in the model system through translations. In the case of the cationic SAM, one of the centered ligands is replaced by the cation of the target molecule. Finally, a random rotation with angle Φ over a *z*-axis (the gold surface is on an *xy*-plane) is performed for each of the substituted molecules to obtain different initial orientations within the ensemble of ligands. Each axis contains the S atom of the corresponding organic molecule that is being rotated. This is performed using the *z*-rotation matrix **R**_**z**_:
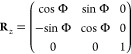
7When all the components are properly treated, they are put together, and, using the tleap utility of AmberTools21,^105^ the parameter and the coordinate files are generated.

## 4 Results and Discussion

**Figure 3:**
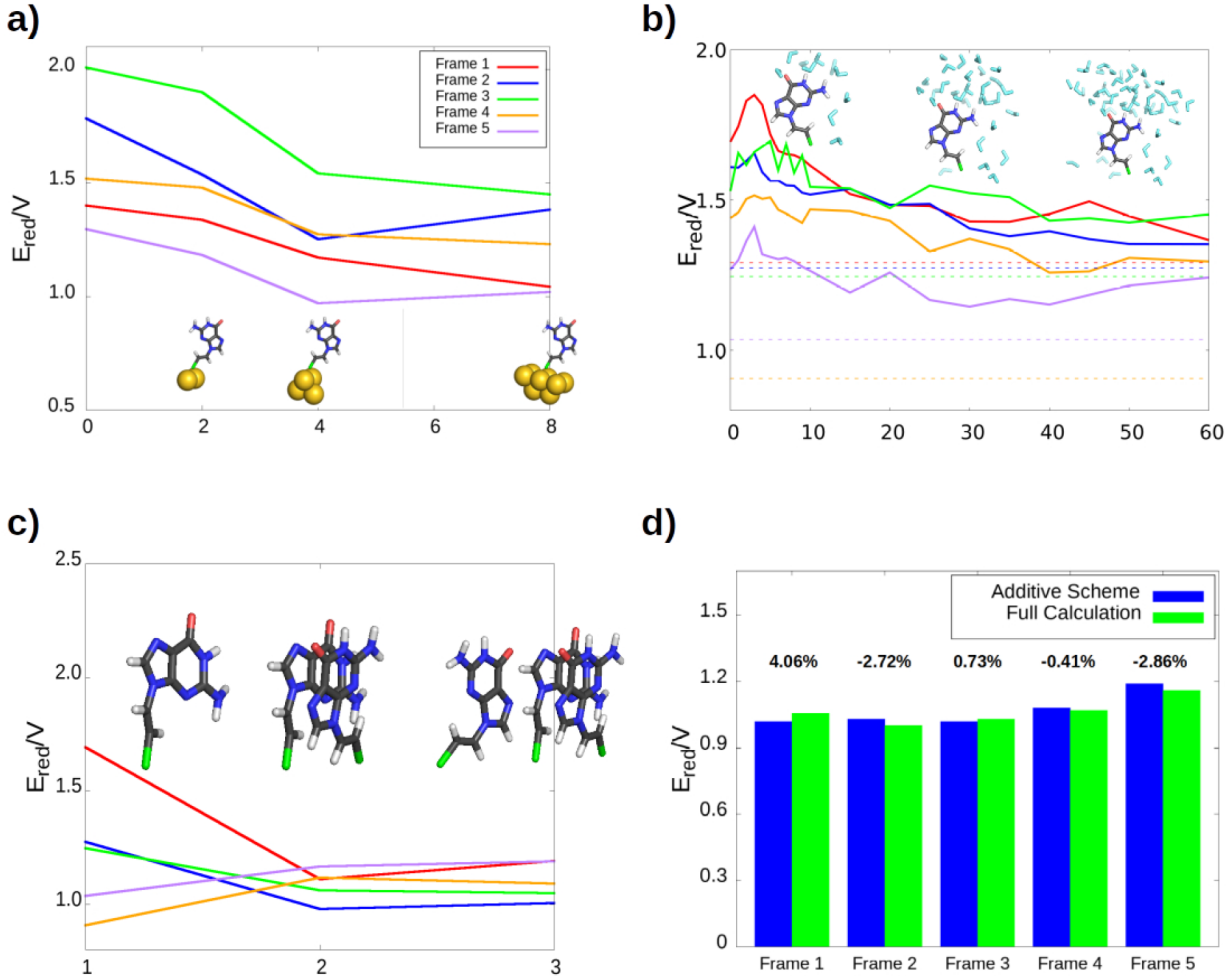
Variation of the one-electron reduction potential with an increasing number of (a) Au atoms, (b) water molecules, and (c) organic molecules (nucleobase + linker) in the QM region. Each color line represents a single snapshot obtained from the MD trajectory. Panel (b) also shows the values of the reduction potential as dashed lines when the COSMO implicit model is used as a solvent. (d) Validation of the additive scheme, compared to a full QM/MM calculation (see text).

The system under study (see Figure 1b) is relatively large from the computational point of view (more than 6000 atoms) since the model considers the metal substrate, a SAM formed by the guanine and linker molecules, and water molecules as the solvent. This implies that the potential energy surface of the system presents different local minima that can be populated at room temperature and that must be considered when computing the redox properties. Thus, the first step of the theoretical protocol was to run classical MD simulations to explore the potential energy surface. Then, the one-electron reduction potential was computed for several snapshots selected from the dynamics by hybrid QM/MM and QM/MM/continuum computations, where the QM region was treated at the DFT level. The smallest part of the system that should be described quantum mechanically is one of the nucleobases and its linker. We will refer to this smallest QM region as the reference ligand (RL). However, the environment surrounding this moiety can significantly affect the value of the reduction potential and, thus, it could also be necessary to consider additional parts of the system to be included in the QM region. It is possible to distinguish three different components in the SAM: the ensemble of nucleobases with their linkers, the gold surface, and the solvent. The effect of including each of this components in the QM region on the one-electron reduction potential is investigated here (see Figure 3).

### 4.1 Convergence of the QM Region

We start by analyzing the effect of the Au atoms on the reduction potential of the system. Since the linker that connects the nucleobase and the metallic substrate is small, the guanine/gold nonbonded interactions can be strong and influence the electronic properties of the nucleobase. Therefore, the evaluation of the ligand–surface interactions is important. Figure 3a shows the effect of introducing an increasing number of Au atoms in the QM region for five geometries randomly selected from the dynamics. The first two Au atoms considered are the closest ones to the S atom of the RL unit located on the upper layer of the metal substrate. The following two Au atoms to be treated quantum mechanically are the nearest ones to the S atom, which are located on the lower layer of the Au surface. The last four Au atoms were chosen to be the adjacent atoms to the two first selected Au atoms on the upper layer of the surface. The results revealed that there is a significant decrease of the reduction potential when an increasing number of Au atoms are included in the QM region. Although full convergence is not completely reached in Figure 3a, the energy difference when going from four Au atoms to eight Au atoms in the QM region is not too large for any of the five analyzed geometries and, thus, increasing the QM size with more than four Au atoms is not worth considering the huge computational cost that it requires. If the attention is focused on the location of Au atoms of the QM region along the surface, the lower shell of the surface still modifies the redox properties of the ligand. This might be a consequence of the coupling between the electronic clouds of the upper and lower layers of the metal and its effect on the S–Au bond, which, in turn, modifies the reduction potential of guanine. In fact, it seems that the inclusion of the Au atoms of the lower layer is even more relevant than the upper one for some of the geometries (e.g., frames 3 and 4 in Figure 3a). In terms of convergence and computational cost, including four Au atoms in the QM region seems to be the best option.

From a parallel perspective, the way that the solvent is described can influence the computed value of the one-electron reduction potential of the guanine nucleobase embedded in the SAM. The solvent can be described by explicit and implicit models in QM/MM and QM/continuum calculations, respectively.^123^ To analyze the behavior of the reduction potential with explicit solvation, the same five geometries used above were also analyzed. Specifically, an increasing number water molecules (up to 60) were included in the QM region together with the RL, while the remaining water molecules were described by the TIP3P force field (see Figure 3b). In general terms, the QM/MM potential experiences an increase in its value when the first 10 water molecules are considered in the QM region. Then, when going from 10 to 20 QM water molecules, the potential decreases and, finally, becomes constant for larger QM region sizes. Therefore, the description of 20 water molecules in the QM layer is enough to reach a converged value of the reduction potential. In addition, a description of the solvent through the continuum COSMO solvation model was also considered.^63,64^ Despite their limitations related to the lack of atomic resolution, QM/continuum schemes imply three clear advantages over a QM/MM description:

(i) The QM region becomes smaller, since all the solvent molecules are described by the continuum model; since a lower computational cost is required, other components can be included in the QM region.
(ii) The COSMO approach is a polarizable solvent model in which both the solvent and the solute polarize each other in a self-consistent manner, leading to a better description of the solute/solvent interactions.
(iii) A continuum model such as COSMO represents an average over multiple configurations of the solvent, not an explicit one, and, therefore, a smaller number of snapshots is needed to obtain converged results for an ensemble of geometries.

The dashed lines displayed in Figure 3b show the values of the computed reduction potential for each of the five geometries analyzed above using COSMO to describe the solvent. As can be seen, when implicit solvation is employed the reduction potential is smaller than that for explicit solvation, especially for the frames 4 and 5 in Figure 3b. This can be a consequence of the better description of the interactions and the averaging nature of the model, as mentioned above. It is important to mention that a polarizable QM/MM model, or any QM/MM model where the QM region includes a large number of water molecules, might potentially provide the same or higher accuracy than a QM/COSMO model. However, these explicit solvent models would require a high computational cost, because a large number of geometrical configurations would be needed to obtain an average solvent description as the one provided by continuum models.

The third component of the device is the organic monolayer (SAM) formed by the guanine and linker molecules (the ligands). Because of the aromatic character of the ligands, with large electronic π-systems, it will not be surprising that the interactions between them can modify the value of the one-electron reduction potential of the system. Thus, the addition of neighbor molecules to the RL moiety in the QM layer can be of paramount importance for the determination of the potential. The same procedure as those explained above for metal atoms and water molecules was also applied for ligands (see Figure 3c). It is important to highlight that the positive charge of the cationic species during the QM/MM calculations within the Marcus theory was restrained to be in the guanine of the RL. This means that charge delocalization among different ligands has not been taken into account, since it is out of the scope of this work. However, delocalization effects could be relevant and need to be addressed in future research. Figure 3c shows that the addition of the closest ligand to the RL has a big impact on the value of the reduction potential. This is clearly due to a response to the interactions between π-systems of adjacent molecules. The addition of a third ligand into the QM region does not affect the value of the potential and, thus, the inclusion of only one ligand in the QM layer, in addition to the reference one, is enough to account for these interactions.

### 4.2 Additive Scheme

The inspection of Figures 3a–c provides the ideal size of the QM region: two ligands, four Au atoms, and 20 water molecules. However, these results also give rise to a conflict. All these elements in the QM region would lead to highly expensive calculations that are not worthwhile to afford. To alleviate the computational cost, the solvent will be described by COSMO, since, as discussed above, it represents a good choice to reduce the number of calculations and to improve the solute/solvent bulk interactions. Despite the application of the continuum solvent model, performing QM/MM/COSMO calculations where the QM region is formed by two ligands and four Au atoms (and the MM region by the remaining ligands and Au atoms) is still computationally unfeasible, especially considering that hundreds of geometries must be taken into account to obtain converged results. Therefore, an alternative protocol with lower computational cost must be set. In this context, it was tested whether the effect of the individual components of the biosensor to the reduction potential can be considered additive or not. If this is the case, the one-electron reduction potential for the largest QM region (two ligands and four Au atoms) can be written as the sum of the potential for a model where only one ligand composes the QM region plus gold and ligand effects, which are computed by using reduced QM regions (one ligand and four Au atoms for a set of calculations and two ligands for the other):
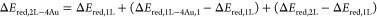
8The calculation of the left-hand term of eq 8 is computationally demanding. Thus, the reliability of this additive protocol will be evaluated by taking as reference QM/MM calculations where the QM region is formed by only two Au atoms and two ligands. However, once the additive scheme is shown to be correct (see below), the right-hand side of eq 8 (including four Au atoms in the QM region) will be applied to compute in a more accurate way the potential of the biosensor. Therefore, for the purpose of the evaluation of the additive scheme, eq 8 converts to eq 9:
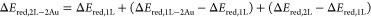
9The subscripts *i*L and *i*Au refer to the number of ligands and gold atoms considered in the QM region, respectively. Figure 3d compares the additive scheme (right-hand terms of eq 9) with the so-called “full QM calculation” (left-hand term of eq 9). The potentials obtained from both sets of calculations were pretty similar, giving evidence that the additive protocol is valid. Differences between potential values for each considered geometry were almost negligible. In all the cases, the relative error of the additive potentials, with respect to the full calculation, was <5%. Therefore, the additive scheme was applied to reduce the QM region size and the computational cost in the following calculations where a large ensemble of snapshots is considered.

### 4.3 One-Electron Oxidation Potential

In the next step, the following procedure was performed to compute the one-electron oxidation potential of the biosensor, taking into account conformational sampling. First of all, classical MD simulations were run for both the neutral and the localized monocationic versions of the SAM, in which only one nucleobase molecule is positively charged. After reaching the equilibrium, 200 geometries were randomly fetched from the trajectory. For each geometry, the three reduction potentials of the right-hand side of eq 8, using different QM region sizes, are computed by applying the Marcus theory, as previously done.^58^ In all the cases, the positive charge was restrained to be located on the guanine molecule of the RL. As mentioned above, solvent effects were included with the COSMO model, and the remaining components of each situation were described by MM point charges, giving rise to a multilayer QM/MM/COSMO scheme. Finally, applying eq 8 and averaging over the 200 snapshots, the one-electron reduction potential of the system was then determined. These calculations were performed for three different biosensors which differ in the linker that anchors the guanine to the gold surface: thioethane, thioethene, and thioarene. In order to choose the linkers, a set of static calculations were performed for simplify models formed by guanine, different linkers, and COSMO solvent (see the Supporting Information). The one-electron reduction potential of these models was calculated using the direct static approach established in a previous work.^58^ The range of the potentials obtained for the different linkers was small: 0.99–1.28 V. Thus, there is not a big influence of the linker bonded to the free guanine nucleobase in solution. The question here is whether this dependence increases or not when the ligand is anchored into a gold surface forming a SAM, where an organic environment and a gold surface are also interacting with the RL. This was investigated for three linkers of different nature: thioalkane, thioalkene, and thioarene.

**Figure 4:**
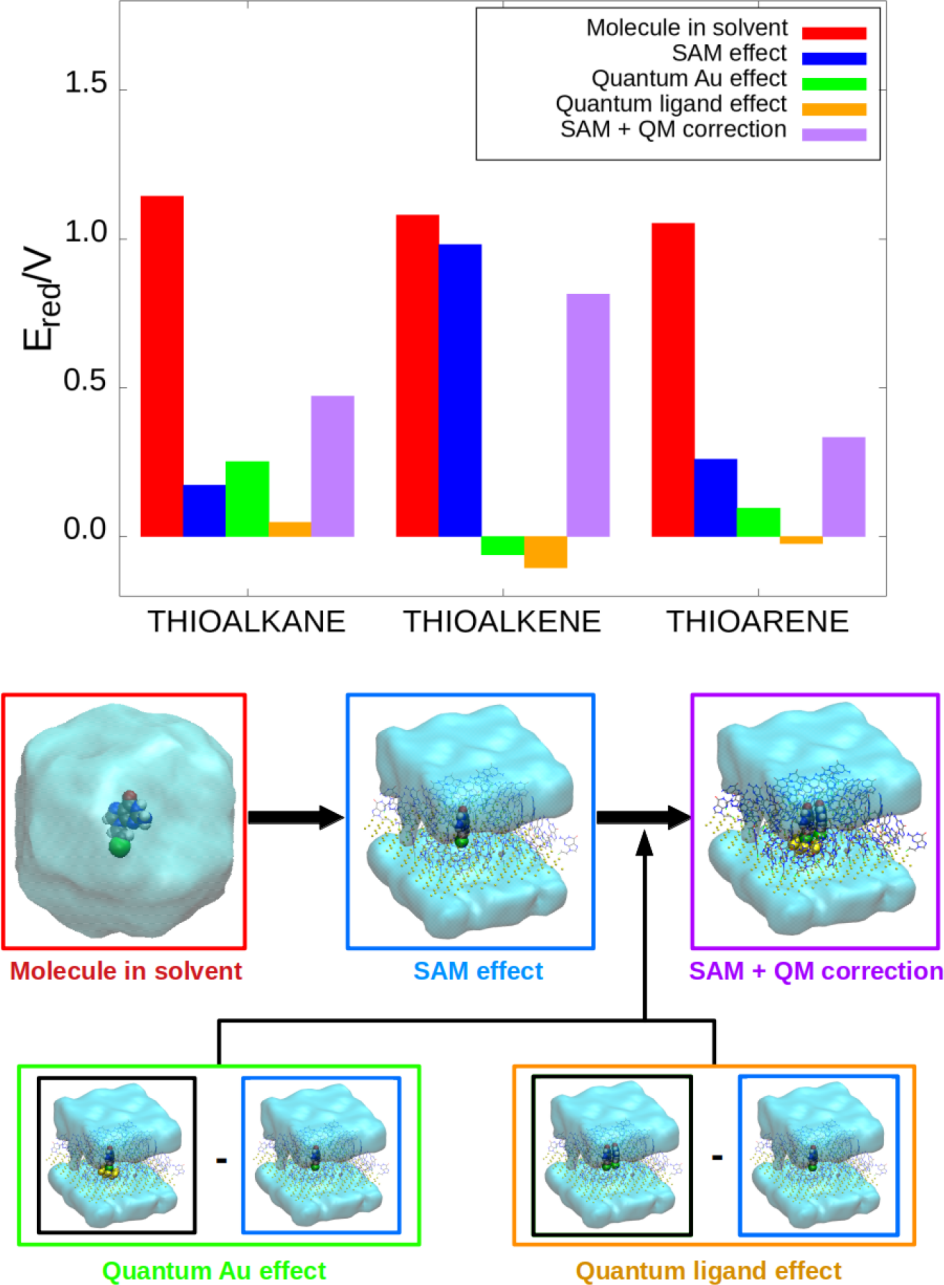
One-electron reduction potential of a guanine residue when it is free in water solution (red bar), it is placed on a SAM which is described by a force field (blue bar), (iii) the additive scheme is applied (purple bar). The variation in the reduction potential due to the quantum contributions to the additive scheme of the gold (green bar) and ligand (orange bar) moieties are also shown. The schematic representation below the plot shows graphically the process to apply the additive scheme (eq 8). [Color code for the atoms and solvent: sulfur, green; carbon, gray; nitrogen, blue; oxygen, red; hydrogen, white; gold; and the solvent, aquamarine.]

Figure 4 shows the one-electron reduction potentials obtained for each of the three organic species in both situations: a free molecule in an aqueous solvent (red bars) and the molecule as part of a solvated SAM (purple bars). The second situation was obtained using the additive scheme, as described above. As previously mentioned, it can be seen that when the three species are free in solvent, their one-electron reduction potentials are similar: the difference between the most oxidant and the most reducer species is <0.1 V (see also the Supporting Information). Consequently, the linker does not affect the redox properties of the guanine residue significantly when the nucleobase is in solvent. However, this situation changes when each of the organic ligands are embedded into a SAM. The most susceptible system to oxidation, which is the SAM whose linker is a thioarene group, shows a one-electron reduction potential almost 0.5 V lower than the SAM with the thioalkene linker. Therefore, the presence of an environment—refereed to the gold surface and the organic part of the SAM— plays an important role in the redox properties of a single molecule. Even when the additive scheme is not applied and only one ligand is described at a quantum-mechanical level of theory, the differences in their redox properties are clearly evident (blue bars). In fact, the contributions added from the introduction of Au atoms and other ligands in the QM region generally modify in small quantities the values of the redox potentials. This means that the effect of the environment on the reduction potential of the biosensor is already described in a reasonable way by a classical force field, and the introduction of part of the environment in the QM region is needed only to quantitatively refine the value of the potential.

**Figure 5:**
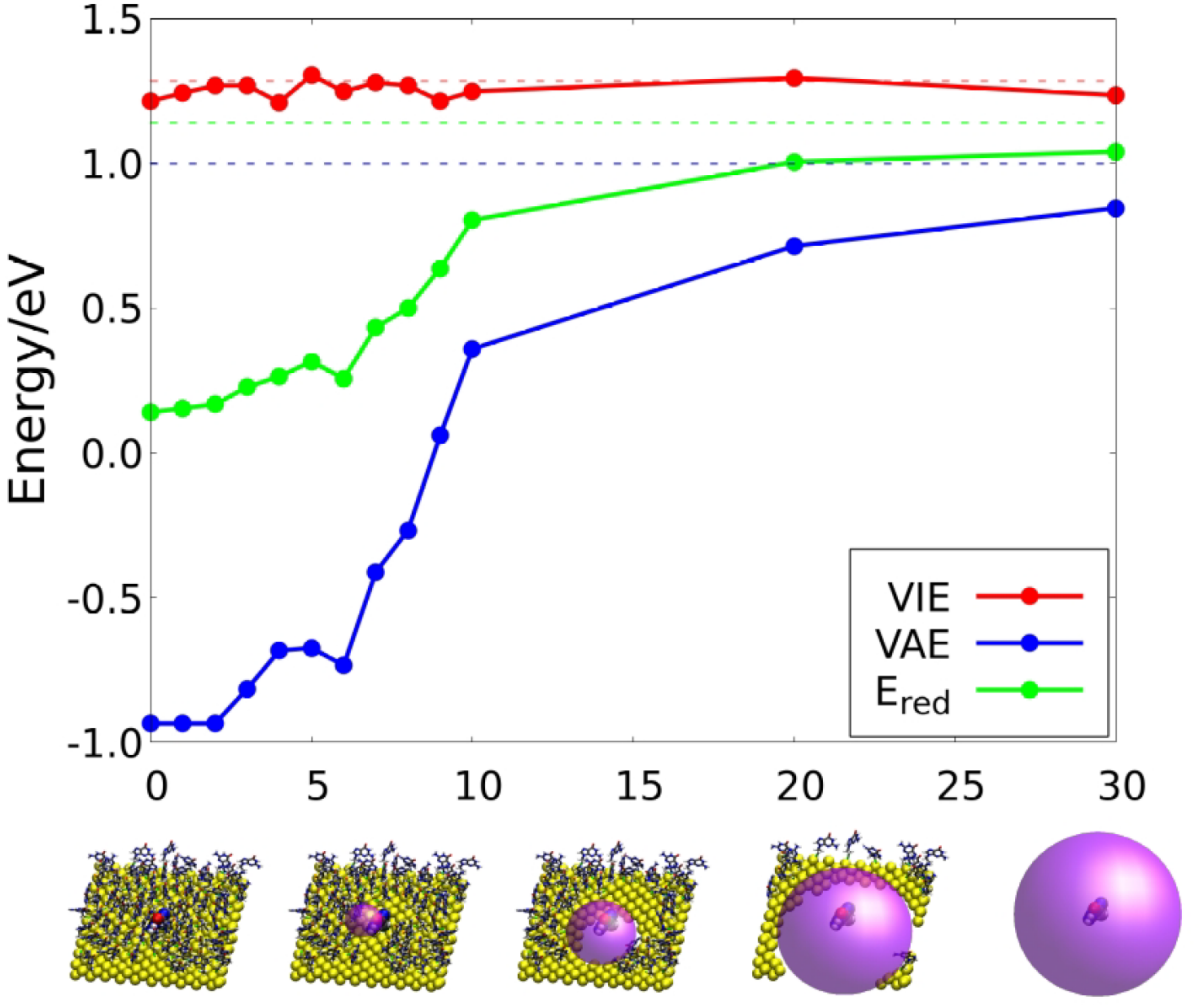
Schematic representation of the calculations performed to study the decrease of the one-electron reduction potential as the environment is taken into account closer to the reference ligand. The solvent is always described using COSMO. The vacuum bubble generated between the classical charges and the QM region is represented by the purple sphere. [Color code: gold, yellow; nitrogen, blue; oxygen, red; carbon, gray; hydrogen, white; and sulfur, green.]

The role of the environment can be analyzed in more detail in Figure 4 by comparing the variation of the potential when going from aqueous solvent (red bar) to the SAM environment (purple bar). The one-electron reduction potential drastically decreases in the SAM. Specifically, the potentials of the SAM with the thioalkane, thioalkene, and thioarene linkers decrease 0.97 V, 0.10 V, and 0.79 V, respectively, with respect to the molecule in solvent. This means that the reducer character of the guanine–linker complex increases due to the electrostatic interactions with the other components of the SAM. In addition, as mentioned above, this behavior is qualitatively reproduced when the environment is described classically (blue bar). This means that the decrease in the potential is caused mostly by the interactions with the organic ligands and not with the Au atoms, since the electrostatic interactions with the latter are predicted to be zero by the force field. In order to corroborate the importance of the electrostatic interactions with the organic region of the SAM, a set of calculations were performed in which the MM charges of the environment were added from a farther radius to a nearer radius, with respect to the RL (see Figure 5, viewing from right to left). We show this analysis for the case of the thioalkane linker because its one-electron reduction potential in the SAM is the most influenced by the SAM, with respect to the molecule in solvent (see red versus blue bars in Figure 4). However, a similar picture is expected for the other two linkers. The results revealed that the vertical ionization energy (VIE) remains constant with the addition of nearer point charges. However, the vertical electron affinity (VEA) decreases considerably as nearer ligands are placed around the RL and, therefore, the reduction potentials also decreases. This trend is also observed in the thioarene case and in a lower extent for the thioalkene linker. Therefore, the electrostatic interactions between the organic fragments of the SAM (nucleobases and linkers) induce an important decrease of the reduction potential of the biosensor.

## 5 Conclusions

In summary, the redox properties for guanine are extremely affected by the molecular environment. When the nucleobase is free in aqueous medium, its potential barely changes by the organic linker. This suggest that the predominant interactions with water, which are similar no matter which liker is bonded to the guanine nucleobase, regulates the redox properties of the system. However, the value of the potential is highly altered when the guanine is part of a SAM with equivalent molecules anchored to a gold surface. First, the introduction of other neighbor ligands decreases the reduction potential, forming a system in which oxidation is more probable to occur. As a result, the redox properties of guanine are enhanced when it is integrated into a SAM, leading to a promising model for a DNA-based biosensor. Second, the nature of the linker that assembles the guanine molecules to the gold surface also affects significantly the one-electron reduction potential. Consequently, it is possible to tune the redox properties of the nucleobases by choosing an appropriate linker, which can be used to improve the selectivity of the device. Finally, this study presents an efficient computational strategy that can be employed to obtain reduction potentials for very large systems at the quantum-mechanical level of accuracy by applying an additive scheme in a computationally affordable manner. Such a theoretical model can help to rationally design new nanobiosensors with tuned redox properties.

## Supporting Information Available

The one-electron oxidation potentials for a large amount of guanine derivatives can be found calculated within the static approach (PDF)
