# Supplementary material for: An Efficient Multilayer Approach to Model DNA-Based Nanobiosensors
Source: J Phys Chem B. 2023 Feb 13;127(7):1513–25. doi: 10.1021/acs.jpcb.2c07225 (PMC9969517; doi:10.1021/acs.jpcb.2c07225)
Supplement: Supplementary file 1 — jp2c07225_si_001.pdf [file jp2c07225_si_001.pdf]

Supporting Information for "An Efficient Multilayer Approach to Model DNA-Based Nano-Biosensors"

Jesús Lucia-Tamudo,[†] Juan J. Nogueira,[*,†,‡] and Sergio Díaz-Tendero[*,†,‡,¶]

†*Department of Chemistry, Universidad Autónoma de Madrid, 28049, Madrid, Spain*

‡*Institute for Advanced Research in Chemistry (IAdChem), Universidad Autónoma de Madrid, 28049 Madrid, Spain*

¶*Condensed Matter Physics Center (IFIMAC), Universidad Autónoma de Madrid, 28049 Madrid, Spain*

E-mail: juan.nogueira@uam.es; sergio.diaztendero@uam.es

One-electron oxidation potentials for free molecules

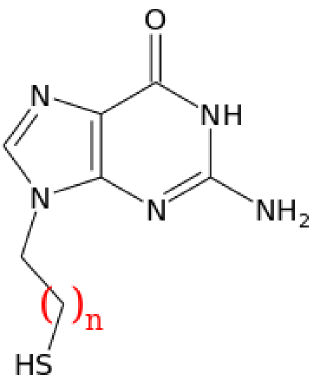

Molecule	
Length of chain	One-electron Oxidation Potential/V
$n = 1$	1.14
$n = 2$	1.17
$n = 3$	1.23
$n = 4$	1.24
$n = 5$	1.24
$n = 6$	1.24
$n = 7$	1.28

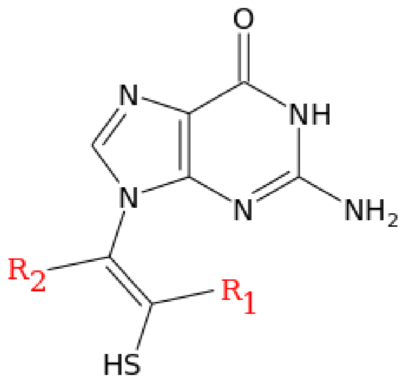

Molecule		
R_1	R_2	One-electron Oxidation Potential/V
$-H$	$-H$	1.08
$-H$	$-OH$	1.05
$-H$	$-NH_2$	0.99
$-H$	$-OCH_3$	1.15
$-H$	$-CH_3$	1.06
$-OH$	$-H$	1.13
$-OH$	$-OH$	1.15
$-OH$	$-NH_2$	1.10
$-OH$	$-OCH_3$	1.15
$-OH$	$-CH_3$	1.07
$-NH_2$	$-H$	1.05
$-NH_2$	$-OH$	1.27
$-NH_2$	$-NH_2$	1.05
$-NH_2$	$-OCH_3$	1.09
$-NH_2$	$-CH_3$	1.10
$-OCH_3$	$-H$	1.17
$-OCH_3$	$-OH$	1.11
$-OCH_3$	$-NH_2$	1.09
$-OCH_3$	$-OCH_3$	1.07
$-OCH_3$	$-CH_3$	1.13
$-CH_3$	$-H$	1.10
$-CH_3$	$-OH$	1.11
$-CH_3$	$-NH_2$	1.11
$-CH_3$	$-OCH_3$	1.10
$-CH_3$	$-CH_3$	1.14

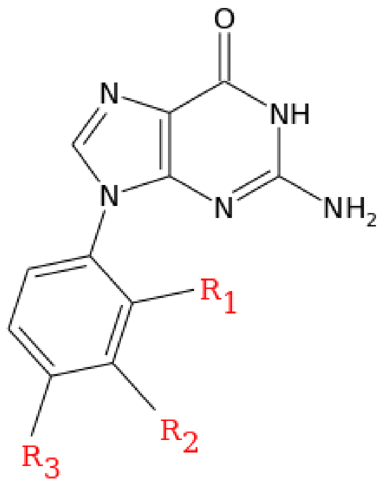

Molecule			
R_1	R_2	R_3	One-electron Oxidation Potential/V
$-SH$	$-H$	$-H$	1.08
$-H$	$-SH$	$-H$	1.05
$-H$	$-H$	$-SH$	1.13

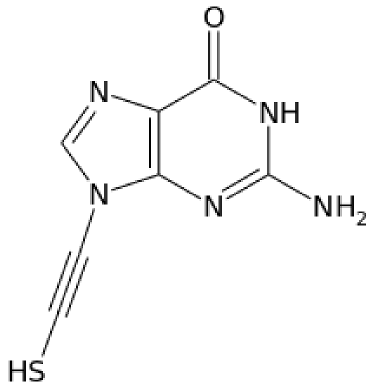

Molecule	
Linker	One-electron Oxidation Potential/V
Unsubstituted	1.08
